# Controllable synthesis of MnO_2_/polyaniline nanocomposite and its electrochemical capacitive property

**DOI:** 10.1186/1556-276X-8-179

**Published:** 2013-04-17

**Authors:** Fanhui Meng, Xiuling Yan, Ye Zhu, Pengchao Si

**Affiliations:** 1 Key Laboratory for Liquid–solid Structural Evolution and Processing of Materials, Ministry of Education, School of Materials Science and Engineering, Shandong University, Jinan 250061, People's Republic of China; 2 School of Chemistry and Chemical Engineering, Shandong University, Jinan 250100, People's Republic of China; 3 School of Chemistry and Bioscience, Ili Normal University, Xinjiang 835000, People's Republic of China

**Keywords:** Interfacial synthesis, Polyaniline, MnO_2_, Composite, Electrochemical capacitor

## Abstract

Polyaniline (PANI) and MnO_2_/PANI composites are simply fabricated by one-step interfacial polymerization. The morphologies and components of MnO_2_/PANI composites are modulated by changing the pH of the solution. Formation procedure and capacitive property of the products are investigated by XRD, FTIR, TEM, and electrochemical techniques. We demonstrate that MnO_2_ as an intermedia material plays a key role in the formation of sample structures. The MnO_2_/PANI composites exhibit good cycling stability as well as a high capacitance close to 207 F g^−1^. Samples fabricated with the facile one-step method are also expected to be adopted in other field such as catalysis, lithium ion battery, and biosensor.

## Background

As a sort of classic conducting materials, polyaniline (PANI) possesses good conductivity with specific organic characters that metal cannot match, which has attracted a lot of attentions for its wide applications in capacitance, sensors, ultrafast nonvolatile memory devices, and chemical catalysis [1-5]. MnO_2_ has been widely studied as a promising environmentally benign transition metal oxide for sensor, catalyst, lithium battery, and electrochemical capacitor [6-9]. In the quest for superior performance, aniline has been polymerized in combining with other materials to obtain a promising performance, in which PANI/graphene, PANI/carbon nanotube, PANI/Au electrode, and others have been successfully synthesized [1,2,10]. Meanwhile, hybrid composites consisted of MnO_2_, and other materials have also been fabricated to improve their behaviors in battery or supercapacitor [11-15]. In particular, the structures of MnO_2_/PANI have been constructed with different methods, and the synergistic effect of MnO_2_ and PANI has been demonstrated in supercapacitor and catalysis toward H_2_O_2_ oxidation or organic dyes [16-20]. Considering the catalyst-size dependent reaction selectivity and agglomeration involved in nanostructures and specific nanoscale architecture, the big challenge for high-efficiency and outstanding features is still the controllable synthesis of uniform structures [21,22].

With respect to PANI synthesis, chemical and physical methods have been recommended [5,23-31], in which the facile interfacial polymerization is a highly flexible approach without any templates [3,23,24]. The oxidant and reducing agent are separated in the aqueous and organic solutions, while the redox reaction can occur at the interface. As far as the products are removed into the bulk solution, new polymerization can happen at the interface while secondary growth of PANI are prevented, in which both the shape and size of the products can be controlled. In addition, synthesis of MnO_2_ via reducing the compounds containing MnO_4_^−^ and MnO_4_^2−^ has been extensively used due to its simpleness and low cost. During that procedure, the pH of KMnO_4_ solution plays a critical role in the intermediate oxidation state and finally the products:

(1) $${\mathrm{MnO}}_{4}^{-}{+2H}_{2}O+3e{=\mathrm{MnO}}_{2}{\left(s\right)+4\mathrm{OH}}^{-}$$

(2) $${\mathrm{MnO}}_{4}^{-}{+8H}^{+}+5e{=\mathrm{Mn}}^{2+}{+4H}_{2}O$$

At a high pH, MnO_2_ is the main product while Mn^2+^ is the final product at a low pH.

Recently, due to the depleting of fossil fuels and the severe environmental problems caused by burning fossil fuels, supercapacitors with large-power density and long-time cycling have attracted attentions of many researchers [25,26]. As low-cost and easily obtained materials, the capacitive properties of MnO_2_, PANI, and MnO_2_/PANI composites have been widely studied [27-29].

In this work, we utilize the above mechanism to deliberately synthesize a series of MnO_2_/PANI composites with controllable morphology and uniform size by means of the interfacial polymerization and adjusting the pH of solutions. In the synthesis, monomer aniline and KMnO_4_ are used as reducing agent in organic solution and oxidant in aqueous solution, respectively. PANI and MnO_2_/PANI are prone to diffusing into the aqueous phase because they are hydrophilic in the doped salt forms [3,23,24]. In the composite, PANI is expected to allow uniform MnO_2_ particle dispersion and convenient electron transfer. In the present study, the formation mechanism and the electrochemical capacitive performance of the composites have been investigated.

## Methods

### Preparation of MnO_2_/PANI

Aniline was firstly distilled under reduced pressure. Then, 0.6 mL of aniline was dissolved in benzene (10 mL) solution, while 0.2 g of KMnO_4_ was dissolved in the solution (20 mL) with 1 M ClO_4_^−^ as the doping anion (we used HClO_4_ as the source of ClO_4_^−^). The organic solution was added into aqueous solutions slowly, and the mixture was kept overnight until the reactions conducted completely. The products were then washed with ultrapure water and centrifuged twice to remove residual benzene and KMnO_4_. Finally, the products were dried in the air for the latter use.

### Preparation of the electrode

The composites were mixed with acetylene black (15 wt.%) and dispersed in 0.5 mL of anhydrous ethanol solution by sonication for 5 min. The mixtures were then cast onto a polished glassy carbon electrode and fasten with 2 μL of nafion ethanol solution (1% V/V). The electrodes were dried in the air for latter testing.

### Characterization

The morphology of the sample was characterized by scanning electron microscopy (SEM, JSM-6700 F, JEOL Ltd., Akishima-shi, Japan) at an accelerating voltage of 10 kV. Transmission electron microscope (TEM) micrographs are taken with a JEOL2100 TEM (JEOL Ltd., Akishima-shi, Japan) operating at 200 kV. X-ray diffraction (XRD) patterns were collected using X-ray powder diffraction (XRD, Bruker D8 Advance X-ray diffractometer, Bruker AXS, Inc., Madison, WI, USA; Cu Kα radiation *λ*=1.5418 Å) at a scan rate of 0.02 s^−1^. Fourier transform infrared spectroscopy (FTIR) analyses were carried out using a Vertex 70 FTIR spectrophotometer (Bruker AXS, Inc., Madison, WI, USA).

A CHI 760C electrochemical workstation (CHI Instruments, Austin, TX, USA) was used to collect electrochemical data. All electrochemical experiments were conducted in a three-electrode cell, in which a 1.5×1.5 cm^2^ Pt plate was used as the counter electrode and a saturated calomel electrode was selected as the reference electrode.

## Results and discussion

**Figure 1:**
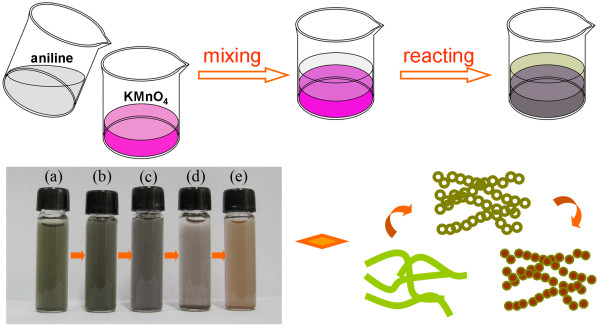
**The schematic of the synthesis procedure and the morphologies of MnO**_**2**_**/PANI composites at different HClO**_**4**_**concentrations**.

The schematic of MnO_2_/PANI fabrication procedure is shown in Figure 1. The reaction commences at the interface of the two solutions immediately as the aniline solution is carefully spread onto the aqueous solution of KMnO_4_. The interfacial polymerization does not terminate until KMnO_4_ or aniline is consumed completely. The products diffuse into the aqueous solution spontaneously due to the doping procedure of the polymers and hydrophilic property of hydrate MnO_2_. The color of the products in different solutions (a to e: 1, 0.5, 0.2, 0.1, and 0 M HClO_4_, respectively, as shown in the inset of Figure 1) turns from green to brown. This color evolvement is attributed to the different components of composites accompanying with the change of PANI-doping degree. The SEM and TEM images, FTIR spectra, and XRD patterns were employed to investigate the components and the formation of the products.

**Figure 2:**
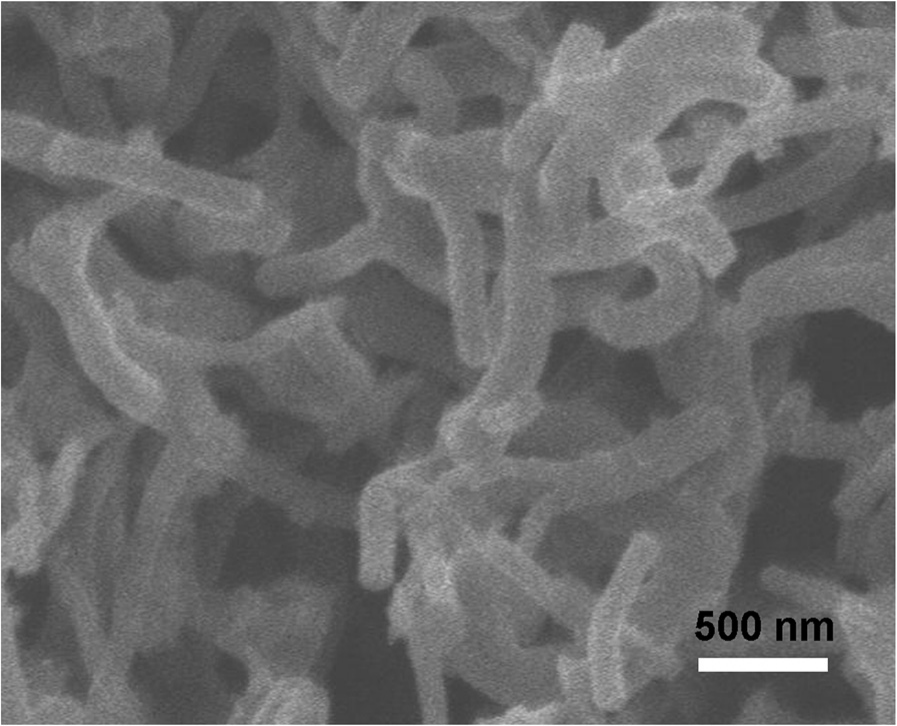
**SEM images of PANI synthesized by interfacial polymerization at 1 M HClO**_**4**_**.**

**Figure 3:**
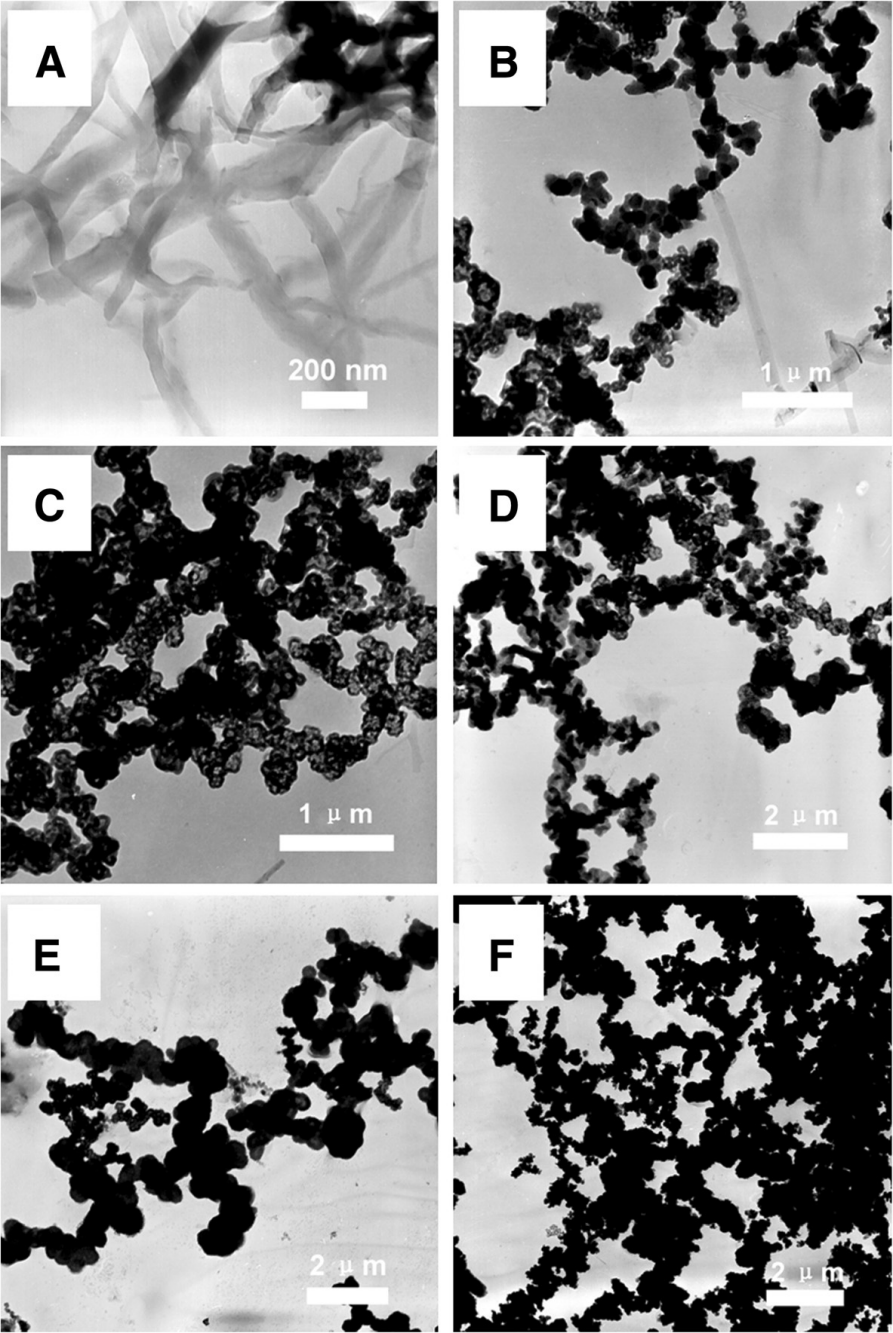
**TEM images of MnO**_**2**_**/PANI composites synthesized at different acid concentrations.** (**A**) 1, (**B**) 0.5, (**C**) 0.2, (**D**) 0.1, (**E**) 0.05, and (**F**) 0 M HClO_4_.

PANI nanofibers form at the interface between the organic and 1 MHClO_4_ aqueous solution. As observed in the SEM image (Figure 2), the diameter and length of the nanofibers are around 100 to 200 nm and over 1 μm, respectively. Additionally, it reveals that the nanofibers are twisted and networks are formed by random interconnection, which agrees with the previous reports [3,23,24]. To indicate the evolvement of the samples' morphologies with the changing of acid concentrations, the TEM images of MnO_2_/PANI fabricated at different acid concentrations are collected in Figure 3. As shown in Figure 3A, PANI nanowires synthesized in 1 M HClO_4_ solution is consistent with the SEM result in Figure 2. When the interfacial polymerization is carried out using 0.5 M HClO_4_ (Figure 3B), the conventional nanowire almost disappears. On the contrary, interconnected agglomerating chains appear. In addition, a number of hollow spheres can be observed. Interestingly, when the acid concentration decreases to 0.2 M (Figure 3C), a larger portion of hollow spheres is observed. However, the portion of hollow spheres is decreasing with the decrease of the acid concentrations in the range of 0.1 and 0 M HClO_4_ (shown in Figure 3D,E,F). In this way, we can modulate the sample structures easily by adjusting the pH of the aqueous solution.

An explanation in the procedure of composite fabrication is proposed in our work. Firstly, aniline monomers are polymerized only at the interface of the organic and aqueous phases, so that hydrophilic nanofibers can be separated from the interface and diffuse into the aqueous solution, which prevent the secondary growth and provide space for new nanofiber growing. Additionally, MnO_2_, as an oxidative regent for PANI polymerization, is used as sacrificial materials in forming various PANI structures [31,32]. According to the change of the morphologies (nanofibers, hollow spheres, and solid particles), it is reasonable to assume that the appearance of the intermediate of MnO_2_ is a critical role in the formation of hollow spheres. As illustrated in Equations 1 and 2, for the low-acid concentration (0.5, 0.2, and 0.1 M), there is not enough H^+^ at the interface to resolve the intermediate of MnO_2_ because of the rapid H^+^ consumption in the reaction (Equation 2). In the meantime, the resolution of MnO_2_ restarts while the composite removes from the interface. The consequential reducing reaction of MnO_2_ follows Equation 3 [33]:

(3) $${\mathrm{MnO}}_{2}{+4H}^{+}+2e{=\mathrm{Mn}}^{2+}{+2H}_{2}O$$

In the acid solution of lower concentrations (0.1 and 0 M HClO_4_), MnO_2_ appears both at the interface and the bulk solution, which caused a little portion of or no hollow spheres to obtain. In our study, it is thought that large amount of MnO_2_/PANI composites can be obtained at low-acid concentration, and the MnO_2_ nanoparticles are wrapped by PANI.

**Figure 4:**
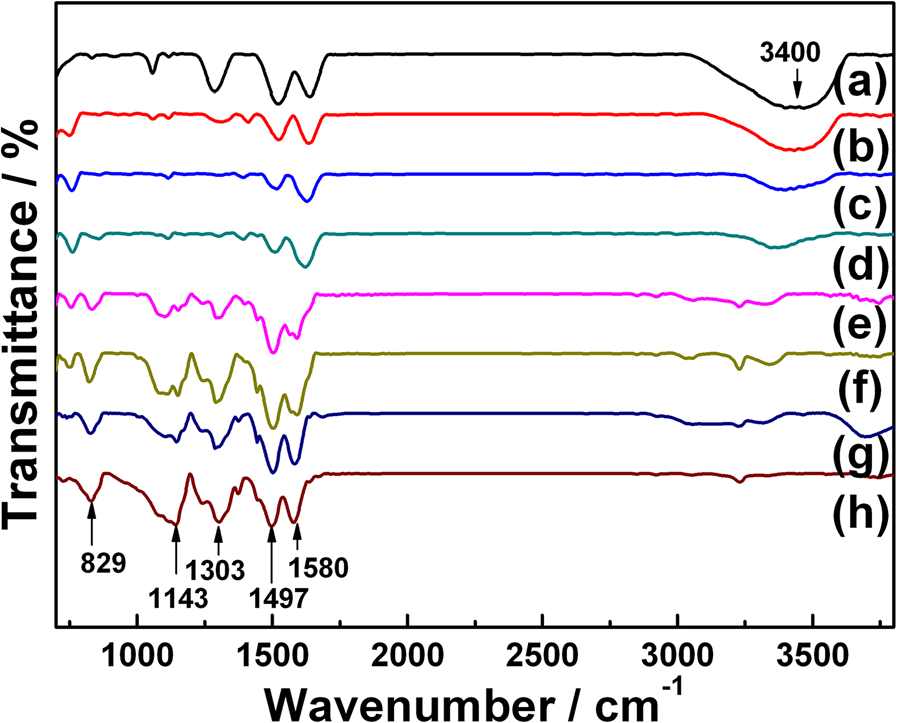
**FTIR spectra of the as-prepared samples.** Curves a to g: MnO_2_/PANI fabricated in 0, 0.02, 0.05, 0.1, 0.2, 0.5, and 1 M HClO_4_, respectively.

FTIR spectra (Figure 4) were measured to identify the component of the different samples. Among peaks assigned to PANI, the characteristic peaks around 1,580 and 1,497 cm^−1^ relate to the stretching vibration of quinoid (−N=(C_6_H_4_)=N-) ring and benzenoid (−(C_6_H_4_)-) ring, respectively. Another main band at 1,303 cm^−1^ can be assigned to the stretching of C-N in -NH-(C_6_H_4_)-NH-. The bands appeared at 1,143 cm^−1^ and 829 cm^−1^ which correspond to the stretching of C-H in-plane and C-H out-of-plane bendings. In addition, the bands of N-H (PANI) and O-H (H_2_O) at 3,230 and 3,400 cm^−1^, respectively, are observed. As noticed, the band near 3,400 cm^−1^ (O-H) is becoming intense with the decrease of the acid concentration, which is attributed to the appearance of hydrate MnO_2_. The above conclusion is proved by the annealing experiments: the band at 3,400 cm^−1^ (O-H) of hydrate MnO_2_ vanished after 500°C heat treatment (Additional file 1: Figure S1). The band near 1,303 cm^−1^ is becoming weaker from curves g to a in Figure 4, which suggests that the doping degree of PANI is changing with the acid concentration. The characteristic bands of curves a, b, and c in Figure 4 shifted right compared with the others, which is ascribed to the effect of MnO_2_ on PANI. It demonstrates that some special interaction exists between MnO_2_ and PANI.

**Figure 5:**
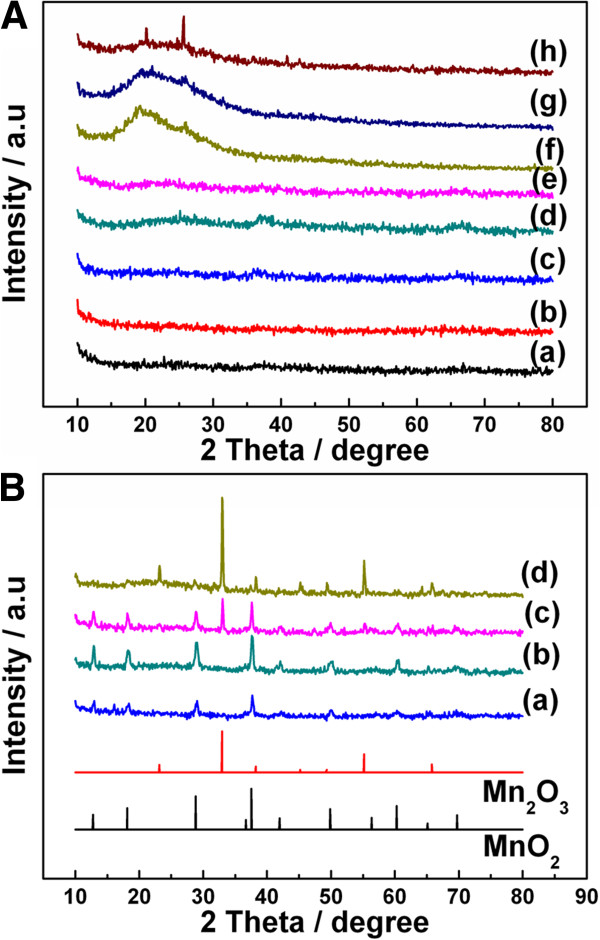
**XRD patterns of the samples.** (**A**) The XRD patterns of the composites, curves a to h: MnO_2_/PANI fabricated in 0.1 M NaOH and 0, 0.02, 0.05, 0.1, 0.2, 0.5, and 1 M HClO_4_, respectively. (**B**) XRD pattern of the samples, curves a to d: annealed MnO_2_/PANI fabricated in 0, 0.02, 0.05, and 0.1 M HClO_4_, respectively.

Due to the ordered and metallic-like property, conducting polymers possess particular crystallinity and orientation. As shown in the XRD patterns in Figure 5A, there are no identified peaks appeared for the products synthesized in low-acid concentrations (curves a to e: 0.1 M NaOH, and 0, 0.02, 0.05, and 0.1 M HClO_4_, respectively), which indicates the products are amorphous. For the products obtained at 0.2 (curve f), 0.5 (curve g), and 1 M HClO_4_ (curve h), two intense XRD peaks 2*θ*≈20 and 25° are observed corresponding to pure PANI according to previous literature [2]. All above results confirm that the crystallized PANI can be formed at higher acid concentrations in this work.

To further analyze the components at different acid concentrations, the samples were treated at 500°C (at which MnO_x_ is crystallizing and PANI will be burned). The products obtained at 1, 0.5, and 0.2 M HClO_4_ were burned out with no solids left, which indicates that there is no MnO_2_ generating at such acid concentrations. Contrary to higher acid concentration, the solid residue of the products obtained at 0.1, 0.05, 0.02, and 0 M HClO_4_ turned black. The FTIR spectra of the heat-treated composites fabricated in 0.1, 0.05, 0.02, and 0 M HClO_4_ were measured (Additional file 1: Figure S2). The characteristic FTIR spectra bands of PANI vanish after heat treatment, which confirms that PANI has been pyrolyzed after heat treatment.

The XRD patterns of the samples after heat treatment are shown in Figure 5B. The XRD patterns of the composite obtained in 0 (curve a) and 0.02 M HClO_4_ (curve b) can be indexed to *α*-MnO_2_ crystal structures [34]. Meanwhile, different XRD peaks are observed in Figure 5B (curves c and d), indicating the heat-treated product obtained in 0.1 M HClO_4_ is Mn_2_O_3_ and the heat-treated product obtained in 0.05 M HClO_4_ are MnO_2_ and Mn_2_O_3_. The results show that for as-prepared samples, Mn_2_O_3_ phase is increasing with acid concentration. It is reported that the phase of manganese oxides is changing with temperature, and MnO_2_ may transform to suboxide Mn_2_O_3_ at 500°C to 900°C [33,35-38]. The reductive matters such as CH_3_OH, CH_4_, and CO were studied as reductions for the phase transforming of MnO_2_ to Mn_2_O_3_, and the mechanism was also suggested [34,39]. Therefore, we assume that the reductive matters generated during PANI decomposition procedure assists the transformation of MnO_2_ to Mn_2_O_3_. Additionally, the aggravating degree of phase transforming of the heat-treated samples could be attributed to the increasing proportion of PANI in the composites. All the above results indicate that the MnO_2_ generated in the polymerization of PANI process at low-acid concentration has a great effect on the formation of the hollow structure at higher acid concentrations as an intermediate.

In this work, the electrochemical performance of the composite was evaluated. The capacitance of MnO_2_ is generated by the charge transferring among multivalent Mn element (Mn^2+^, Mn^3+^, Mn^4+^, and Mn^6+^) [35], while PANI endures doping/dedoping companying with the redox process of PANI:

(4) $$[{\mathrm{PANI}}^{0}{]+A}^{-}\to [{\mathrm{PANI}}^{+}{]\;A}^{-}+e^{-}$$

(5) $$[{\mathrm{PANI}}^{+}{]\;A}^{-}+e^{-}\to [{\mathrm{PANI}}^{0}{]+A}^{-}$$

**Figure 6:**
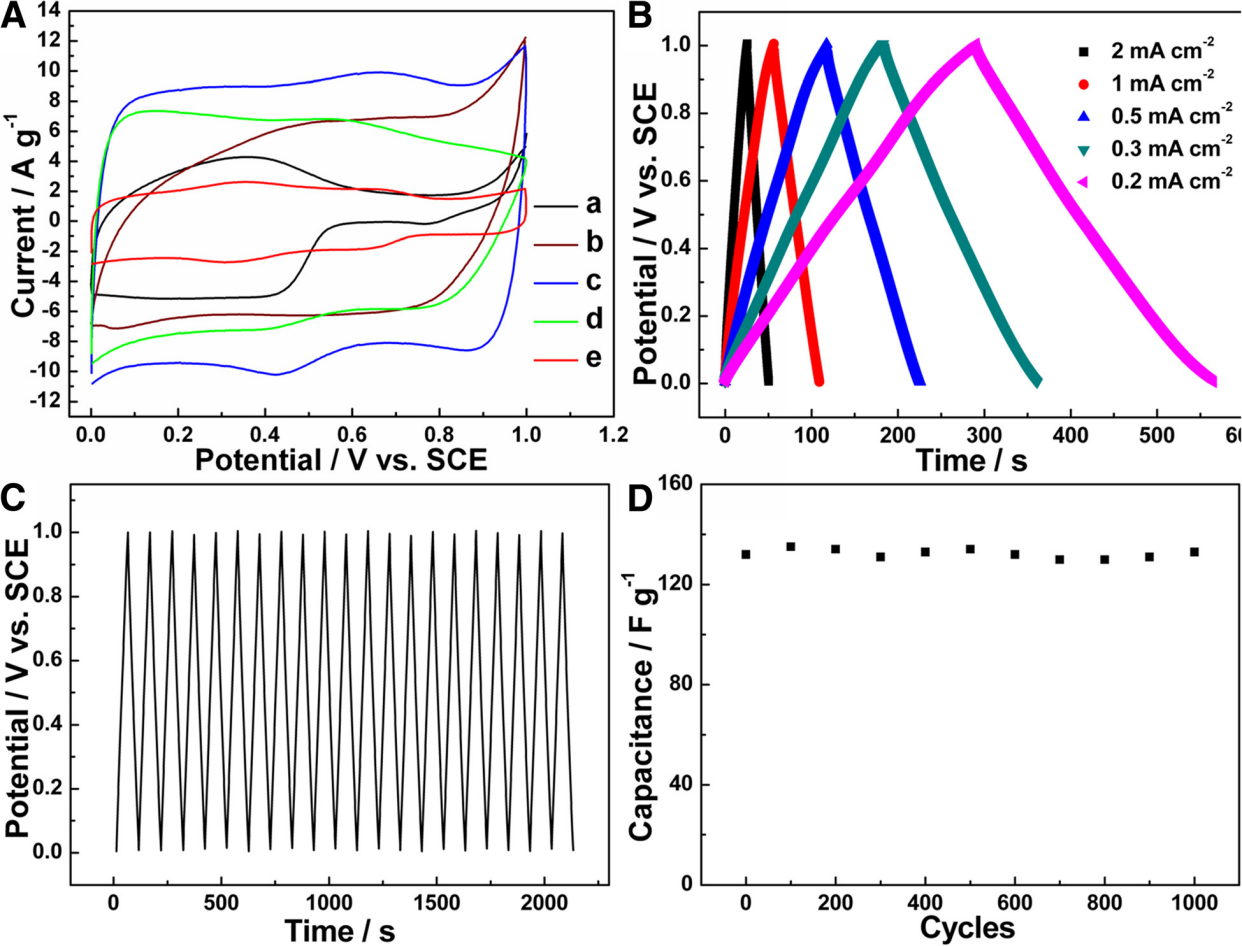
**CV and charge–discharge curves of the composites.** (**A**) CV curves of the as-prepared samples in 0.1 M HClO_4_solution at 50 mV s^−1^, curves a to d: MnO_2_/PANI fabricated in 1, 0.05, and 0.02 M HClO_4_, and 0.1 M NaOH, respectively. Curve e: 500°C-treated MnO_2_/PANI fabricated in 0.02 M HClO_4_. (**B**) Charge–discharge curves of the MnO_2_/PANI composite in 0.1 M HClO_4_ solution at different current densities. (**C**) First 20 cycles of charge–discharge curves for the MnO_2_/PANI composite at the current density of 1 mA cm^−2^ (**D**) Dependence of capacitance of the MnO_2_/PANI composite on the charge–discharge cycles at the current density of 1 mA cm^−2^.

Cyclic voltammetry (CV) curves of the composites are shown in Figure 6A. CV curves of as-prepared PANI nanofibers/MnO_2_ crystallines are comparable with pure PANI and MnO_2_, respectively. The rectangle-like shape of CV curve suggests that MnO_2_/PANI fabricated in 0.02 M HClO_4_ has an ideal capacitive characterization. Additionally, the rectangle-like shape potential region of MnO_2_/PANI (curve c) is relatively larger compared with that of the crystallized MnO_2_ (curve e) and PANI (curve a). The capacitance *C*_*CP*_ can be estimated according to the equation: *C*_*CP*_ = (*Q*_*a*_ + *Q*_*c*_)/(2 × *ΔV*), where *Q*_*a*_, *Q*_*c*_, and *ΔV* are indicative of the anodic and cathodic charges of CV and the potential region of CV, respectively. The capacitances of the samples in curves a to e are 80, 45, 207, 143, and 46 F g^-1^, respectively. The capacitance of MnO_2_/PANI (curve c) is larger than that of PANI (curve a) and MnO_2_ (curve e). The extended ideal capacitive potential region and larger capacitance of MnO_2_/PANI composite are possibly due to the synergistic effect between the core of MnO_2_ and the shell of PANI [32,35,40].

The charge–discharge curves of MnO_2_/PANI fabricated in 0.02 M HClO_4_ were measured at various current densities (shown in Figure 6B). The E-t plots show symmetry, which indicate the reversible charge–discharge process of the MnO_2_/PANI composite. The specific capacitance of the sample can be calculated via the equation: *C*_*CP*_ = *i*/|*dE*/*dt*|, where |*dE*/*dt*| is estimated from the slope of the discharging curves. The capacitance of the composite at 2, 1, 0.5, 0.3, and 0.2 mA cm^−2^ achieves 159, 161, 170, 174, and 168 F g^−1^, respectively. Additionally, the discrepancy of the largest composite capacitance values estimated from discharging and CV curves is lower than 20%, which suggests the high credibility of both techniques.

The stabilities of the samples were tested with 100 CV scan cycles (Additional file 1: Figure S3). After 100 cycles, the CV curves of PANI change obviously and the capacitances decreased largely (Additional file 1: Figure S3 A, B). However, with the increase of MnO_2_, the CV curves change a little and even no capacitance decrease is observed (as shown in Additional file 1: Figure S3 C,D,E). Compared with PANI samples obtained at higher acid concentration, MnO_2_/PANI nanocomposites possess noticeable capacitive stability. To investigate the long-term stability of as-prepared MnO_2_/PANI nanocomposites, the charge–discharge test of 1,000 cycles was conducted at 1 mA cm^−2^ in 0.1 M HClO_4_. As shown in Figure 6C (first 20 cycles are shown for clearly observation), the E-t plots are symmetric in shape and have almost no change during the long-term test. From Figure 6D, it can be seen that the discrepancy of capacitance of MnO_2_/PANI during 2,000-cycle test is lower than 5%, and there is no evident capacitance decrease after 1,000 cycles. The stability of the MnO_2_/PANI composite is thought due to the protection of the shield-surrounded PANI and uniform dispersion of MnO_2_ particles, whereby avoiding severe particles conglomeration involved in the charge–discharge process [35,36]. The facile synthesis and ideal electrochemical capacitive performance will probably give the composites a promising prospect in the application of supercapacitors.

## Conclusions

A series of samples including MnO_2_/PANI composites and PANI nanofibers were successfully synthesized by the facile interfacial polymerization. Controllable and uniform synthesis of the composites has been realized by simply adjusting the pH of the solution. The effect of acid concentration and the related mechanism of the formation of the products are investigated. We demonstrate that the intermediate of MnO_2_ plays a key role in forming the hollow structures of PANI. The capacitance of the composite achieves 207 F g^−1^, and the results suggest that the MnO_2_/PANI composites show superior performance over pure PANI or MnO_2_.

## Competing interests

The authors declare that they have no competing interests.

## Authors’ contributions

FM carried out the total experiment and wrote the manuscript. XY participated in the detection of the SEM and TEM. YZ participated in the data analysis. PS participated in the design of the experiment and performed the data analysis. All authors read and approved the final manuscript.

## Supplementary Material

Additional file 1: Figure S1 FTIR spectra of MnO_2_/PANI fabricated in 0.1 M NaOH, 0 HClO_4_, 0.02 M. **Figure S2.** FTIR spectra of polyaniline (curve a) and the composites after heat treatment (curves b to f): MnO_2_/PANI fabricated in 0.1 M NaOH, and 0, 0.02, 0.05, and 0.1 M HClO_4_. **Figure S3.** CV curves of the composites before and after 100 cycles stability tests in 0.1 M HClO_4_ solution at 50 mV s^−1^, (**A-D**) samples fabricated in 1, 0.05, and 0.02 M HClO_4_, and 0.1 M NaOH and (**E**) MnO_2_ obtained by heating MnO_2_/PANI composite fabricated in 0.02 M HClO_4_. Click here for file
