# Supplementary material for: Controllable synthesis of MnO2/polyaniline nanocomposite and its electrochemical capacitive property
Source: Nanoscale Res Lett. 2013 Apr 17;8(1):179. doi: 10.1186/1556-276X-8-179 (PMC3655007; doi:10.1186/1556-276X-8-179)
Supplement: Additional file 1: Figure S1 — FTIR spectra of MnO2/PANI fabricated in 0.1 M NaOH, 0 HClO4, 0.02 M. Figure S2. FTIR spectra of polyaniline (curve a) and the composites after heat treatment (curves b to f): MnO2/PANI fabricated in 0.1 M NaOH, and 0, 0.02, 0.05, and 0.1 M HClO4. Figure S3. CV curves of the composites before and after 100 cycles stability tests in 0.1 M HClO4 solution at 50 mV s−1, (A-D) samples fabricated in 1, 0.05, and 0.02 M HClO4, and 0.1 M NaOH and (E) MnO2 obtained by heating MnO2/PANI composite fabricated in 0.02 M HClO4. [file 1556-276X-8-179-S1.doc]

**Supporting information for**

**“Controllable synthesis of MnO2/Polyaniline nanocomposite and its electrochemical capacitive property”**

Fanhui Meng^1,2^, Xiuling Yan^2,3^, Ye Zhu^2^, Pengchao Si^1,*^

^1^Key Laboratory for Liquid-Solid Structural Evolution and Processing of Materials, Ministry of Education, School of Materials Science and Engineering, Shandong University, Jinan 250061, People's Republic of China

^2^School of Chemistry and Chemical Engineering, Shandong University, Jinan 250100, People's Republic of China

^3^School of Chemistry and Bioscience, Ili Normal University, Xinjiang 835000, People's Republic of China

*Corresponding Author: Associate Professor Pengchao Si, E-mail: [PCSi@sdu.edu.cn](mailto:PCSi@sdu.edu.cn)

Phone: +86-531-88399858; Fax: +86-531-88395011


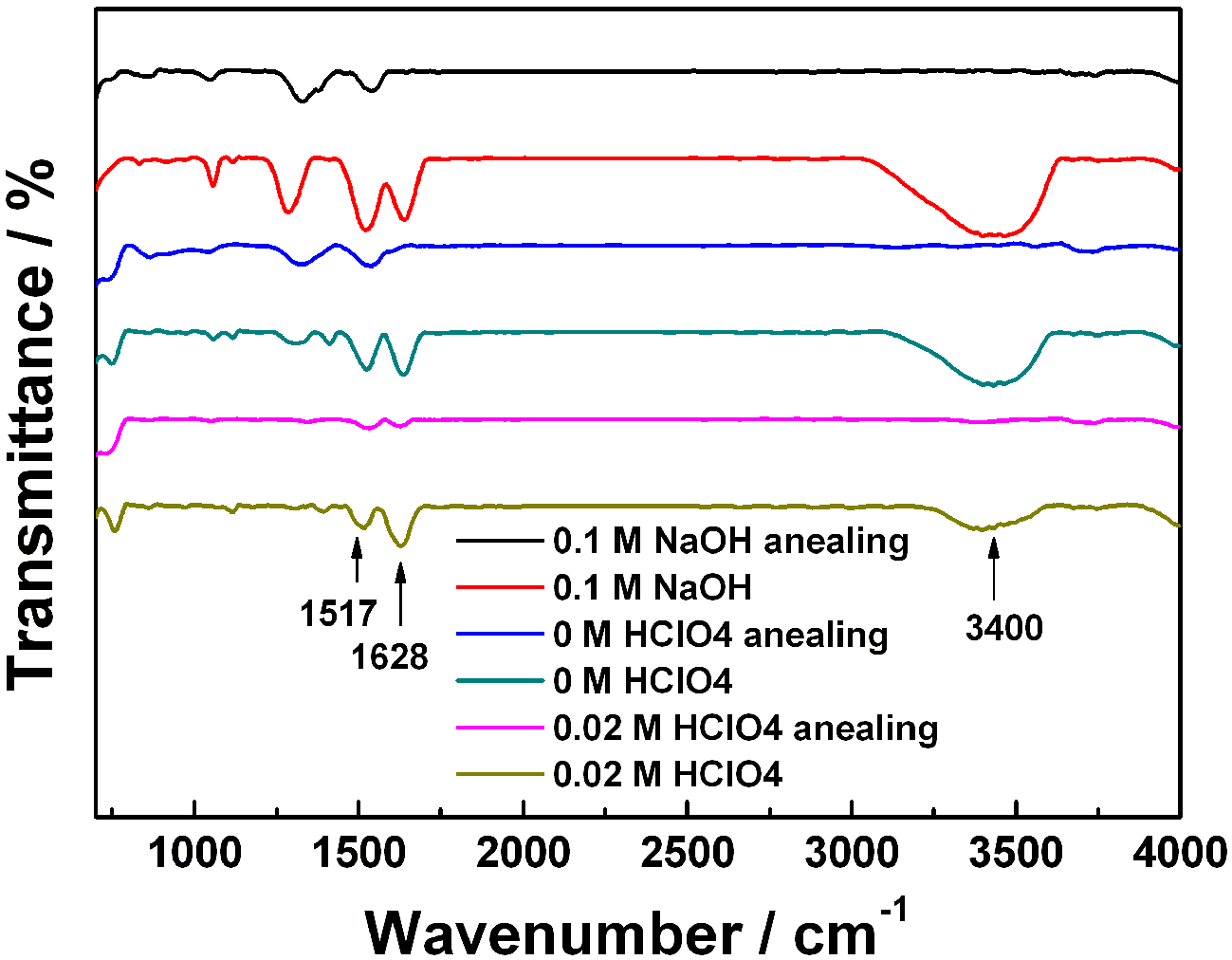


Fig. S1. FTIR spectrums of MnO_2_/PANI fabricated in 0.1 M NaOH, 0 HClO_4_, 0.02 M HClO_4_, before and after annealing treatments.


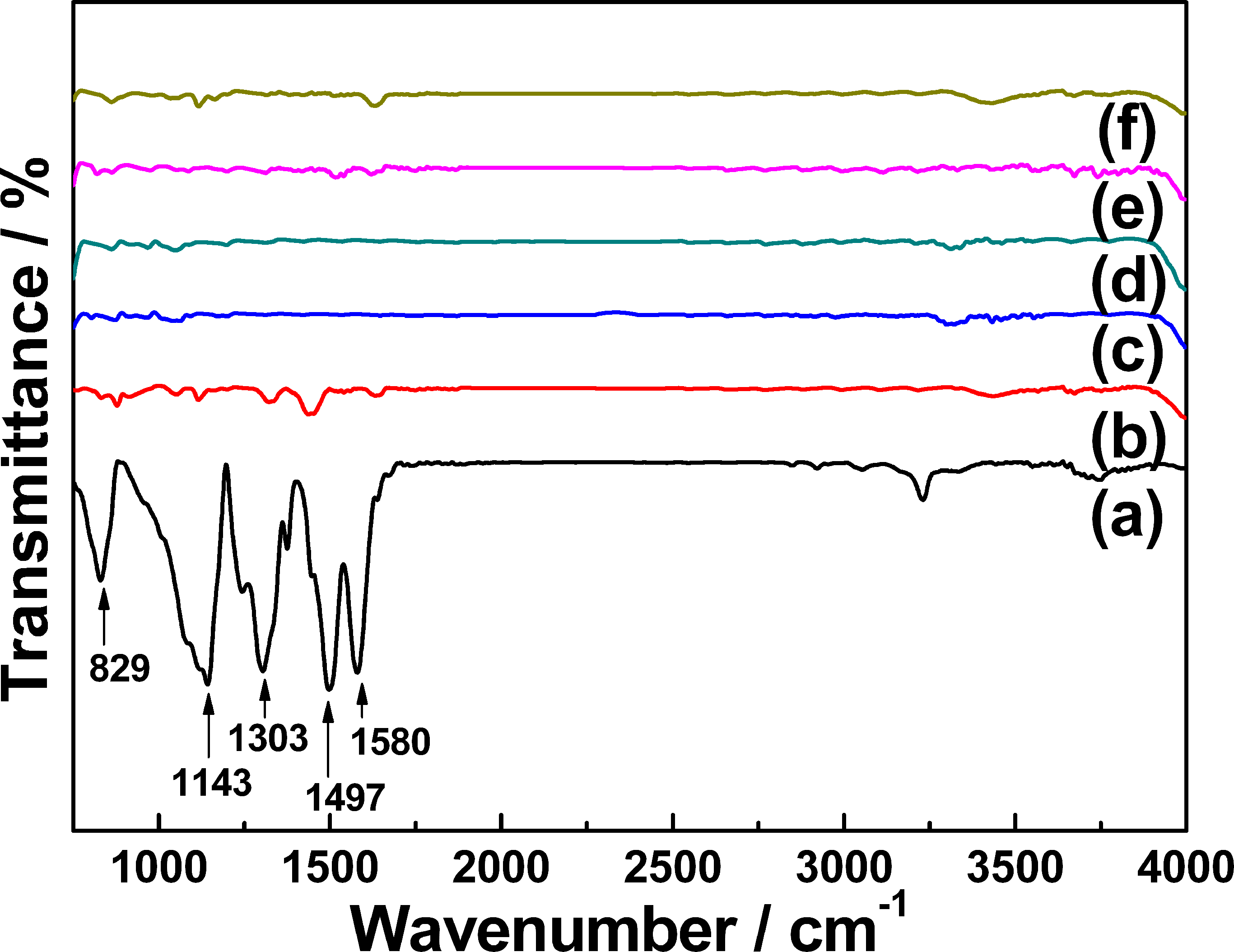


Fig. S2. FTIR spectrums of polyaniline (a)， and the composites after heat treatment (b)-(f): MnO_2_/PANI fabricated in 0.1 M NaOH, 0 HClO_4_, 0.02 M HClO_4_, 0.05 M HClO_4_ and 0.1 M HClO_4_.


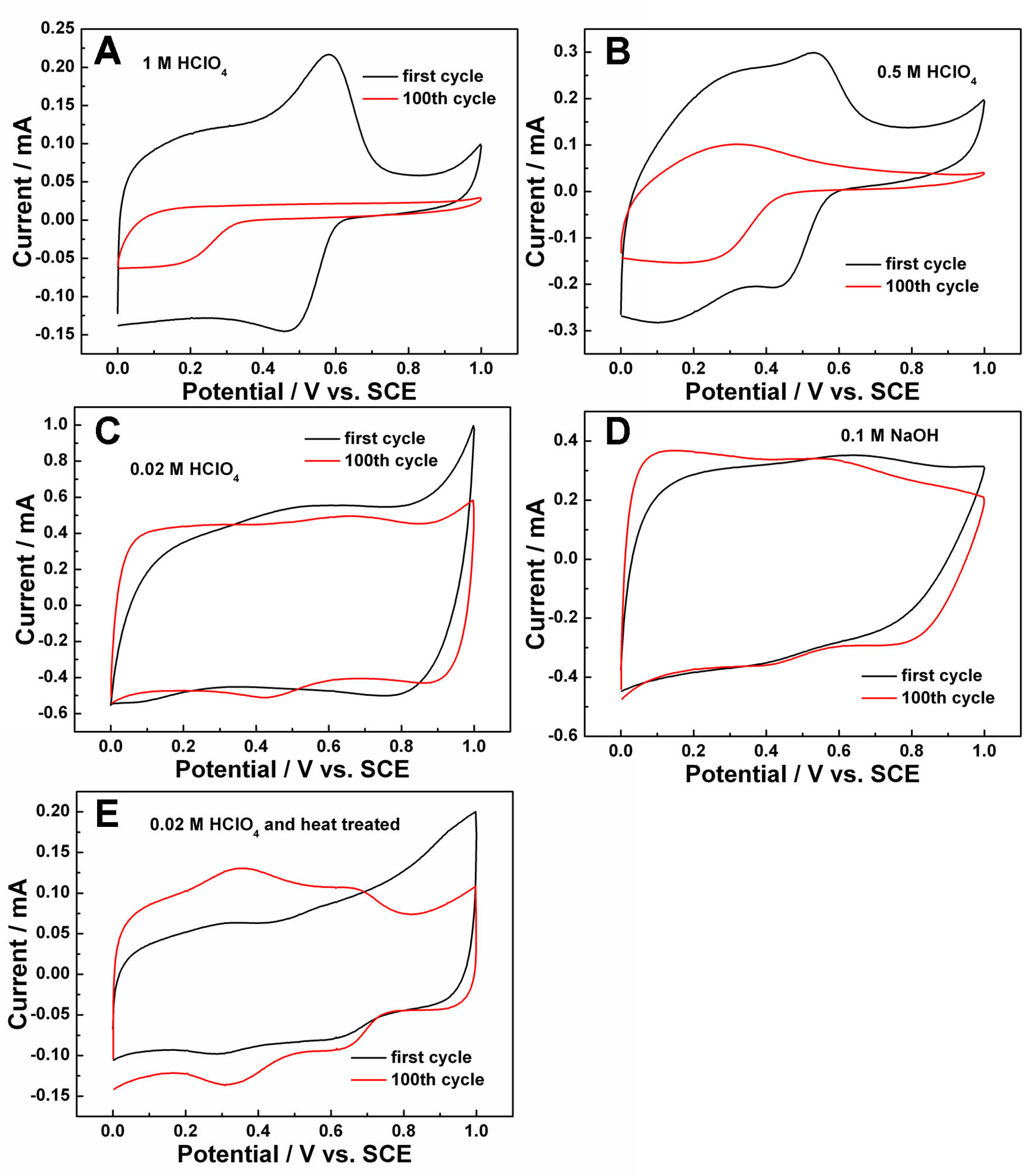


Fig. S3. CV curves of the composites before and after 100 cycles stability tests in 0.1 M HClO_4_ solution at 50 mV s^-1^, (a) – (d) samples fabricated in 1 M HClO_4_, 0.05 M HClO_4_, 0.02 M HClO_4_, 0.1 M NaOH and (e) MnO_2_ obtained by heating MnO_2_/PANI composite fabricated in 0.02 M HClO_4_.
